# The EHEC Type III Effector NleL Is an E3 Ubiquitin Ligase That Modulates Pedestal Formation

**DOI:** 10.1371/journal.pone.0019331

**Published:** 2011-04-26

**Authors:** Heather Piscatelli, Shalaka A. Kotkar, Megan E. McBee, Sureshkumar Muthupalani, David B. Schauer, Robert E. Mandrell, John M. Leong, Daoguo Zhou

**Affiliations:** 1 Department of Biological Sciences, Purdue University, West Lafayette, Indiana, United States of America; 2 Department of Biological Engineering and Comparative Medicine, Massachusetts Institute of Technology, Cambridge, Massachusetts, United States of America; 3 Agricultural Research Service, United States Department of Agriculture, Albany, California, United States of America; 4 Department of Molecular Genetics and Microbiology, University of Massachusetts Medical School, Worcester, Massachusetts, United States of America; Ecole Polytechnique Federale de Lausanne, Switzerland

## Abstract

Enterohemorrhagic *Escherichia coli* (EHEC) O157:H7 causes hemorrhagic colitis and may result in potentially fatal hemolytic uremia syndrome in humans. EHEC colonize the intestinal mucosa and promote the formation of actin-rich pedestals via translocated type III effectors. Two EHEC type III secreted effectors, Tir and EspFu/TccP, are key players for pedestal formation. We discovered that an EHEC effector protein called Non-LEE-encoded Ligase (NleL) is an E3 ubiquitin ligase. *In vitro*, we showed that the NleL C753 residue is critical for its E3 ligase activity. Functionally, we demonstrated that NleL E3 ubiquitin ligase activity is involved in modulating Tir-mediated pedestal formation. Surprisingly, EHEC mutant strain deficient in the E3 ligase activity induced more pedestals than the wild-type strain. The canonical EPEC strain E2348/69 normally lacks the *nleL* gene, and the ectopic expression of the wild-type EHEC *nleL*, but not the catalytically-deficient *nleL*(C753A) mutant, in this strain resulted in fewer actin-rich pedestals. Furthermore, we showed that the *C. rodentium* NleL homolog is a E3 ubiquitin ligase and is required for efficient infection of murine colonic epithelial cells *in vivo*. In summary, our study demonstrated that EHEC utilizes NleL E3 ubiquitin ligase activity to modulate Tir-mediated pedestal formation.

## Introduction

Enterohemorrhagic *Escherichia coli* (EHEC) O157:H7 is an important cause of food- and water-borne illnesses in developed countries and in the world. EHEC O157:H7 infections cause hemorrhagic colitis and can result in potentially fatal hemolytic uremia syndrome [1], [2], [3]. EHEC along with Enteropathogenic *E. coli* (EPEC) and *Citrobacter rodentium* form a group of pathogens called A/E pathogens that are able to colonize the intestinal mucosa and produce characteristic ‘attaching and effacing’ (A/E) lesions. A/E lesions are characterized by effacement of the brush border microvilli, intimate attachment of the bacterium to the plasma membrane of the enterocytes, and the formation of actin-rich pedestals within the host cell beneath the adherent bacteria [4], [5], [6], [7]. Pedestal formation requires virulence-related EHEC proteins to be injected directly into the host cell through a type III secretion system (TTSS) [8]. Two EHEC O157:H7 type III secreted effectors, Tir and EspFu/TccP, are known to be required for pedestal formation [9], [10], [11]. EspM was shown to inhibit pedestal formation via an unknown mechanism [12], [13].

Tir is a bacteria-made receptor that binds bacterial surface protein intimin to facilitate the pedestal formation [14], [15], [16]. Binding to intimin is followed by coordinated events that lead to rearrangement and/or assembly of actin to form pedestals on the host cell surface. The N-terminus of the Tir domain binds several host focal adhesion proteins, including α-actinin, talin and vinculin, and cortactin to which may promote actin networks that support the pedestal [17], [18]. EHEC and EPEC Tir C-termini appear to be modified by serine/threonine phosphorylation upon entry into the host cell [19], [20]. In addition, the C-terminus of EPEC Tir is tyrosine phosphorylated, leading to the recruitment of the host adaptor protein Nck, which in turn stimulates actin assembly [21], [22]. In contrast, EHEC Tir is not tyrosine phosphorylated and does not recruit Nck. Instead, EHEC translocates a second effector, EspF_U_/TccP, which co-localizes with Tir at the site of EHEC attachment. EspF_U_ activates N-WASP by binding to its CDC42-binding domain [9], [10], [23].

Ubiquitination is a reversible posttranslational modification of cellular proteins, in which a 76 amino acid polypeptide, ubiquitin, is attached to the ε-amino group of lysines in target proteins. Ubiquitination is a major player in regulating a broad range of cellular processes, including cell division, differentiation, signal transduction, protein trafficking, and quality control [24], [25]. Ubiquitination involves a multienzyme cascade consisting of classes of enzymes known as ubiquitin-activating enzymes (E1), ubiquitin-conjugating enzymes (E2) and ubiquitin protein ligases (E3). The E3 ubiquitin ligases play pivotal roles in defining the specificity of target proteins to be ubiquitinated. Most E3 ubiquitin ligases belong to two families: one contains the HECT domain and the other possesses the RING finger domain [24]. The HECT family E3 ubiquitin ligases contain a 350-residue region that maintains a strictly conserved cysteine residue that is located approximately 35 residues from the C-terminus [26], [27]. Many pathogenic microbes have developed means to interfere with different stages of ubiquitin pathways to promote their survival and replication. These include SopA, SlrP, SspH1, and SspH2 from *Salmonella* [28], [29], [30], [31], [32]; AvrPtoB from *Pseudomonas syringae* [33], [34], [35]; IpaH 9.8 and IpaH3 from *Shigella flexneri* [31], [36], [37]; and LubX from *Legionella pneumophila* [38].

More than 60 putative EHEC type III effectors have been proposed using the proteomic approach [39] and bioinformatic studies [40]. Previous studies have suggested that the EHEC EspX7 (ECs1560) as an type III effector [40], [41], [42]. EHEC EspX7 is predicted to encode a 782 amino acid peptide and is found on prophage Sp6 [43]. EspX7 homolog is found in the closely related A/E pathogen, *C. rodentium* [44] but not in EPEC E2348/69. We report here that EspX7 is an E3 ubiquitin ligase with C753 being critical for its ligase activity. This E3 ligase activity plays an important role in down-modulating the pedestal formation. We have renamed EspX7 as NleL (Non-Lee-Encoded effector Ligase) to reflect its novel biochemical activity.

## Results

### Self-ubiquitination of GST-NleL

**Figure 1:**
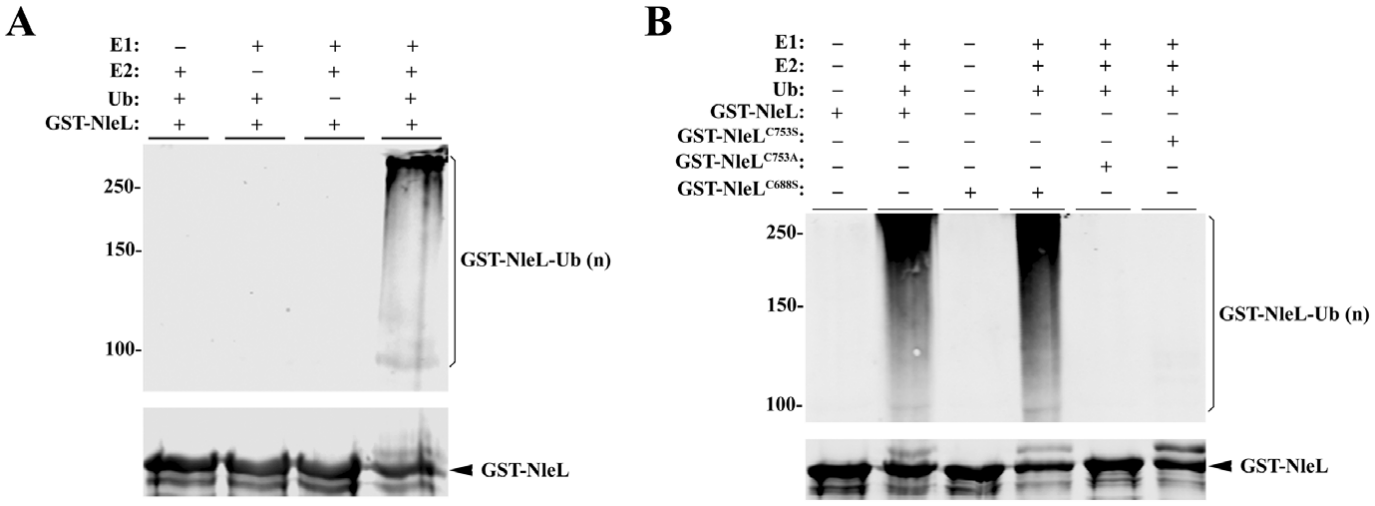
Self-ubiquitination of GST-NleL. (**A**) NleL-mediated self-ubiquitination requires ATP, ubiquitin, E1 and E2. Combinations of ATP, ubiquitin, E1, UbcH5a and GST-NleL^59–782^ were incubated at 35°C for 90 min, and the Western blot was performed using polyclonal anti-ubiquitin antibodies (top) or anti-GST antibodies (bottom). (**B**) NleL C753 residue is required for its E3 ubiquitin ligase activity. Reactions containing ubiquitin, E1 and UbcH5a were incubated with the wild type GST-NleL, GST-NleL^C688S^ or GST-NleL^C753S^ or GST-NleL^C753A^ at 35°C for 90 min. The Western blot was performed using monoclonal anti-ubiquitin antibodies (top) or anti-GST antibodies (bottom).

A study using a bioinformatic approach and validation with various translocation assays has identified multiple translocated effectors in EHEC including the NleL (ECs1560) locus [40]. A sequence comparison and structural studies have identified NleL as a bacterial HECT-like E3 ubiquitin ligase [28], [30], [45]. The substrate and biological function of NleL remains unknown. Auto-ubiquitination is often used in the absence of a physiological substrate to measure the ubiquitin E3 ligase activity, we first tested if GST-NleL has the E3 ligase activity in an *in vitro* auto-ubiquitination assay using E1, E2 (UbcH5a), ATP, and ubiquitin in the presence of purified recombinant GST-NleL^59–782^. Consistent with our previous work [45], poly-ubiquitinated GST-NleL was observed by Western blot when E1, E2 or ubiquitin were added to the reaction (**Fig. 1A**). No ubiquitination of GST-NleL was detected in the absence of E1, E2 or ubiquitin, indicating that each of these components were essential for GST-NleL ubiquitination. Furthermore, the mutant GST-NleL^C753S^ (pZP1658) or the GST-NleL^C753A^ (pZP2129) failed to form the poly-ubiquitination pattern seen with the wild-type GST-NleL in the *in vitro* ubiquitination assay (**Fig. 1B**). To assess the specificity of the cysteine mutation, a substitution mutant targeting C688 (C688S, pZP1657) of NleL was used in the same ubiquitination assay. Western blot analysis shows presence of mono- and poly-ubiquitinated GST-NleL^C688S^ species similar to those observed when the wild-type GST-NleL was used (**Fig. 1B**).

### Mutant NleL^C753A^ is translocated at similar efficiency as the wild-type NleL into mammalian host cells

**Figure 2:**
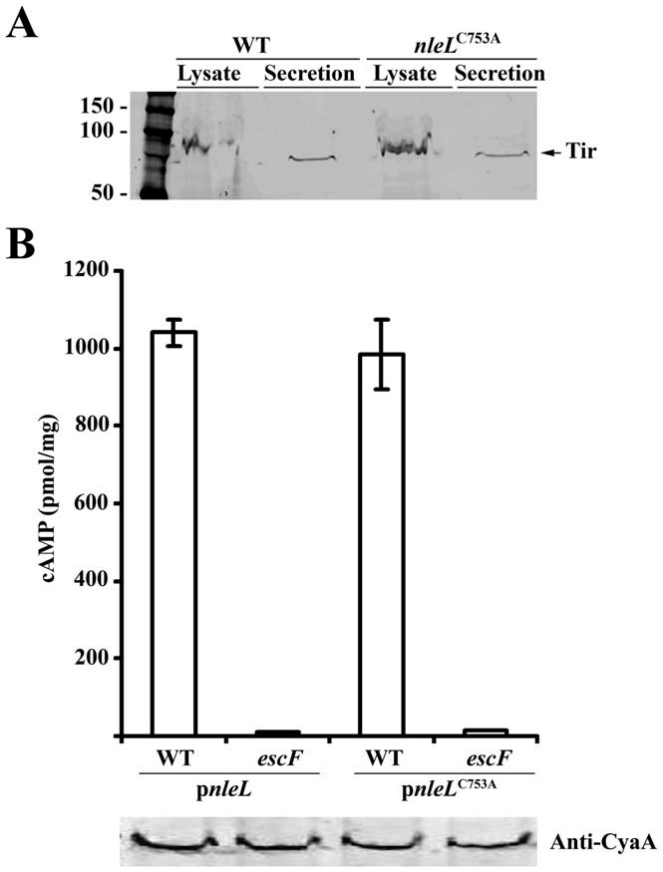
Secretion of Tir and translocation of NleL into HeLa cells. (**A**) The expression and secretion levels of Tir in either the wild-type EHEC or the mutant EHEC expressing the chromosomal catalytically-dead mutant NleL^C753A^ was examined by Western blot. (**B**) Intracellular cAMP levels are an indication of the translocation of CyaA fusion proteins in EHEC WT (ZP250) and EHEC TTSS deficient mutant (*escF*; ZP2251) strains. Cells were infected for 4 hrs, and the adenylate cyclase activity was determined. The data were from three independent experiments, with standard deviations shown as error bars. cAMP values are presented as pmol per milligram of total cellular protein. The expression levels of the NleL-CyaA fusions were found to be similar by Western blot as shown on the bottom panel.

To test whether NleL^C753A^ mutant affected the secretion of other type III effectors, we examined the secretion of Tir, a key player in EHEC-mediated actin pedestal formation upon infection. The expression and secretion levels of Tir in EHEC expressing the chromosomal catalytically-dead mutant NleL^C753A^ (ZP254) was found to be similar to that of the wild type EHEC (**Fig. 2A**). Previous studies have shown that NleL (EspX7) might be a type III EHEC effector [40], [41], [42]. We further tested whether NleL was translocated using an adenylate cyclase (CyaA) reporter system [46]. The CyaA from *B. pertussis* requires host cell derived calmodulin for its activity, hence fusion proteins of CyaA only with proteins that translocate into the host cell have catalytic activity which can be examined by measuring cAMP levels. We compared cAMP levels from HeLa cells infected with either the wild-type strain (ZP250) or a TTSS-deficient EHEC strain (*escF*, ZP251), both harboring the reporter plasmid expressing NleL-CyaA in-frame fusion (pZP1671). Cells infected with the wild-type EHEC strain show approximately 400-fold higher cAMP levels as compared to those infected with the TTSS-deficient strain (**Fig. 2B**). These results confirmed that NleL is indeed an effector protein translocated via the EHEC O157:H7 type III-secretion system.

Similarly, we tested the translocation of NleL^C753A^ using the *cyaA* reporter gene assay. HeLa cells were infected with wild-type EHEC harboring the plasmid encoding either the wild type NleL-CyaA, or the NleL^C753A^-CyaA fusion protein. We found that the cAMP levels were similar to those detected in cells infected with *Salmonella* harboring the wild-type NleL. This indicates that the C753A mutation did not significantly alter the translocation of NleL (**Fig. 2**). Expression of the wild-type and the C753A mutant NleL-CyaA fusion proteins in bacterial whole cell lysates was further confirmed to be at similar levels by Western analysis (**Fig. 2** **, lower panel**).

### E3 ligase activity of NleL down-regulates the EHEC pedestal formation

**Figure 3:**
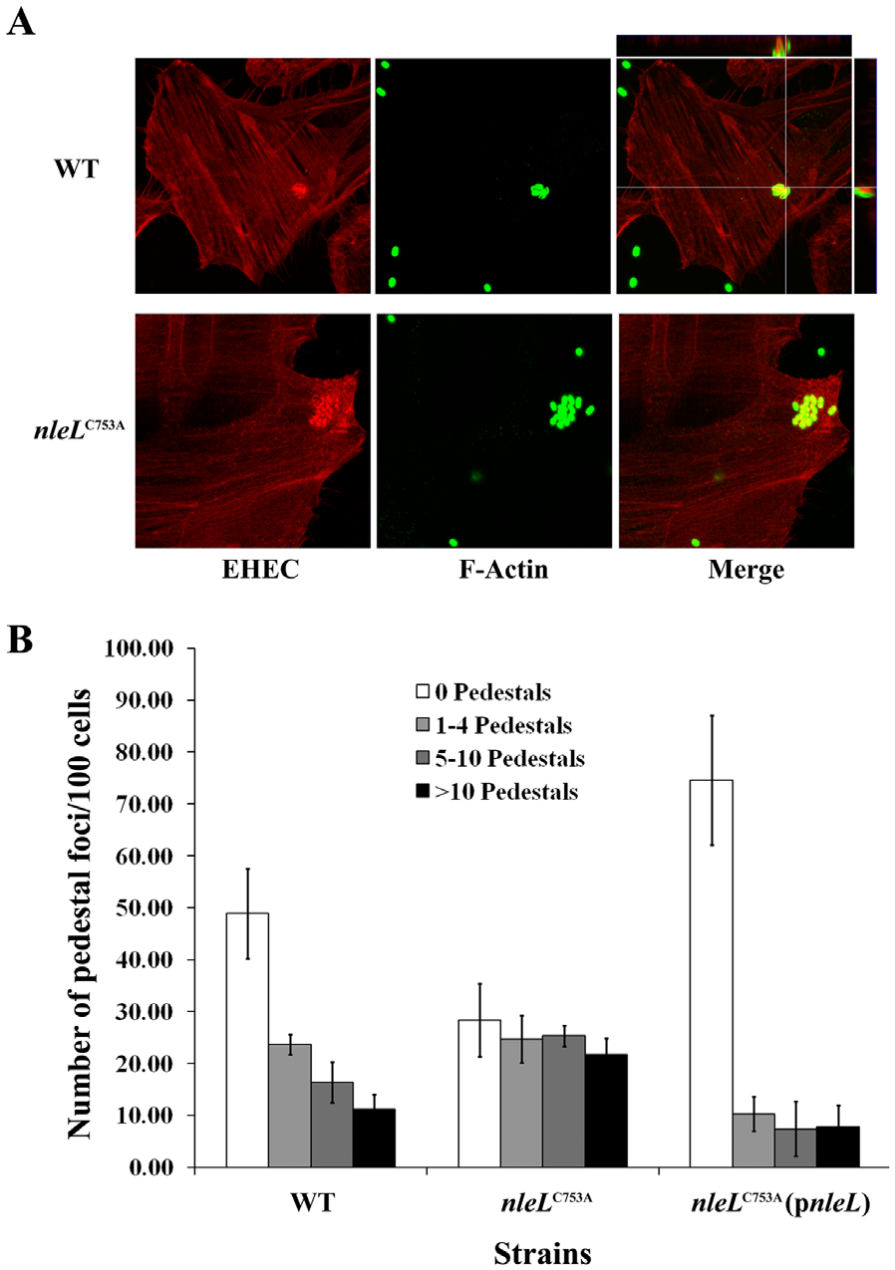
E3 ligase activity of NleL is involved in modulating EHEC pedestal formation. HeLa cells were infected at a multiplicity of infection of 100 with wild type EHEC, *nleL^C753A^*, or *nleL^C753A^* harboring plasmid expressing wild-type NleL. Cells were infected for 6 hours. (**A**) Bacteria were visualized by staining with an anti-EHEC LPS antibody (green). Actin was detected with a Texas Red Phalloidin (red). (**B**) The number of micro-clusters of pedestals on HeLa cells were counted and were grouped into clusters having 1–4 pedestals, 5–10 pedestals and >10 pedestals. Quantitative analysis includes three independent experiments. A minimum of 300 cells were counted from each experiment with standard deviation shown as error bars.

A central function of the EHEC translocated effectors is to promote pedestal formation, which is a hallmark of A/E pathogens [47]. Pedestals are actin-rich structures formed as a direct result of effectors that bring about cytoskeletal rearrangements upon translocation by the EHEC TTSS [23]. Two EHEC effectors, Tir and EspFu are sufficient to induce the formation of pedestals [9], [10], [17], [48], [49]. To test whether the ligase activity of NleL is involved in pedestal formation, an EHEC strain expressing the chromosomal catalytically-dead mutant NleL^C753A^ was used to infect HeLa cells to examine its ability to form pedestals. The number of pedestals formed in HeLa cells infected with ZP254 was increased significantly as compared to those in cells infected with the wild-type ZP250 (**Fig. 3**). For example, the percentage of cells with no pedestals decreased two-fold, and the percentage with more than 10 pedestals increased more than two-fold. The phenotype of ZP254 infected HeLa cells could be rescued by introducing wild-type NleL expressing plasmid (pZP1666) into ZP254. Together, these data indicate that the E3 ligase activity of NleL plays a major role in down-regulating pedestal formation during EHEC infection.

### The E3 ligase activity of EHEC NleL down modulate EPEC pedestal formation

**Figure 4:**
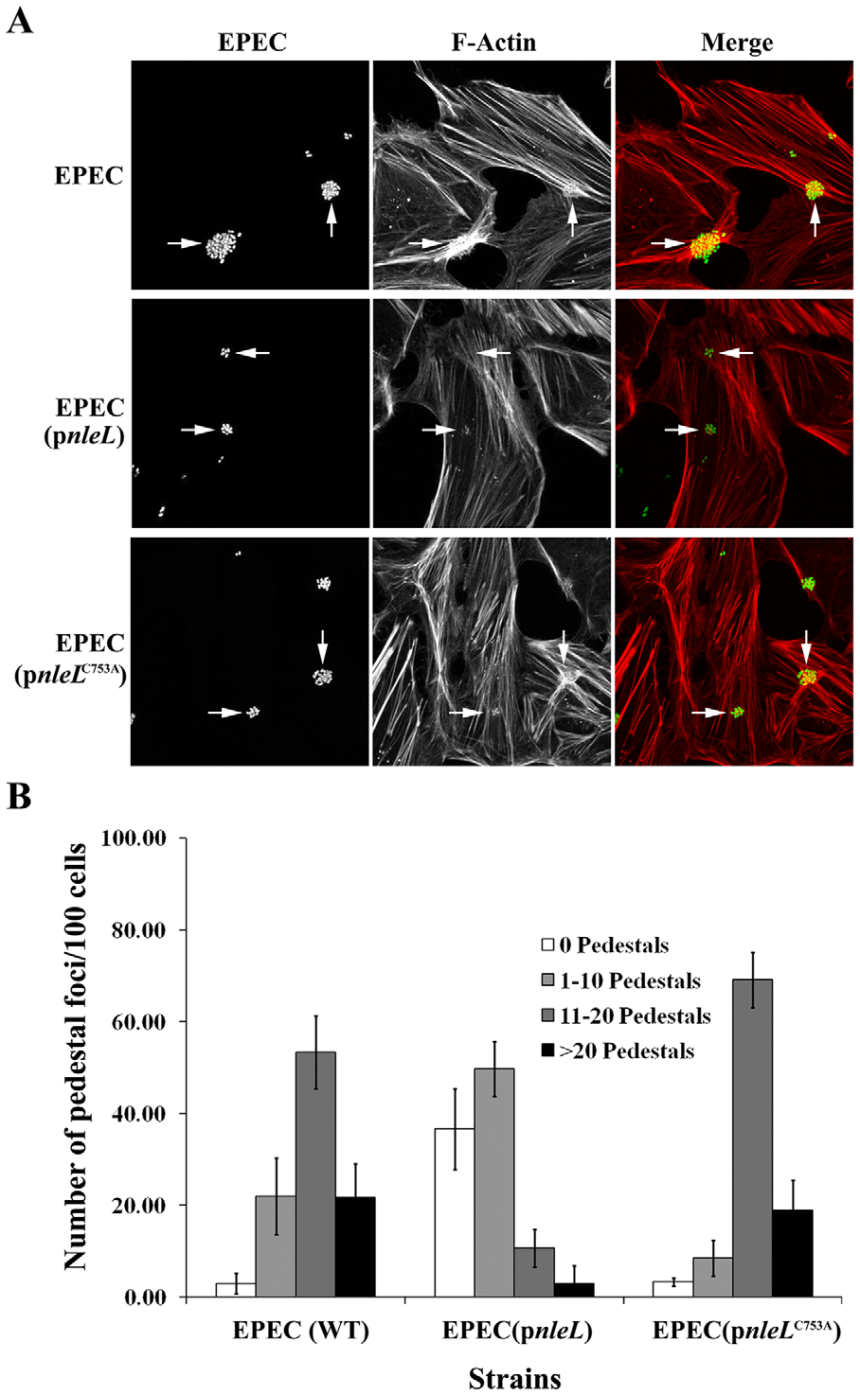
The E3 ligase activity of EHEC NleL down modulates EPEC pedestal formation. HeLa cells were infected at a multiplicity of infection of 100 with wild type EPEC, or EPEC harboring plasmid expressing wild-type EHEC NleL or NleL^C753A^. Cells were infected for 4 hours. (**A**) Bacteria were visualized by staining with an anti-EPEC LPS antibody (green). Actin was detected with a Texas Red Phalloidin (red). (**B**) The number of micro-clusters of pedestals on HeLa cells were counted and were grouped into clusters having 1–10 pedestals, 11–20 pedestals and >20 pedestals. Quantitative analysis includes three independent experiments. A minimum of 300 cells were counted from each experiment with standard deviation shown as error bars.

A genome-wide search of the A/E pathogens revealed that EPEC strains do not harbor the *nleL* gene. We showed that the E3 ligase activity of NleL down modulates the pedestal formation during EHEC infection. Unlike EHEC, canonical EPEC strains do not encode EspF_U_, and generate pedestals by recruiting the host adaptor Nck after phosphorylation of a tyrosine residue in the Tir C-terminus, one that is lacking in EHEC Tir [21], [22]. To determine if, in spite of this mechanistic difference, EHEC NleL also acts to diminish pedestal formation during EPEC infection, we introduced EHEC NleL into the wild-type EPEC strain and assessed their ability to form pedestals. As shown in **Fig. 4**, an EPEC strain expressing the wild type EHEC NleL induced significantly less pedestals compared to that induced by the wild-type EPEC or EPEC expressing the catalytically-inactive NleL^C753A^. For example, the percentage of cells with no pedestals was ∼10-fold higher when wild type NleL was delivered. This result suggests that rather than diminish a process specific to Nck or EspF_U_ the E3 ligase activity of NleL functions to down modulate a common step of pedestal formation by EPEC and EHEC.

### *C. rodentium* NleL is an E3 ligase and is essential for *C. rodentium* virulence in mice

**Figure 5:**
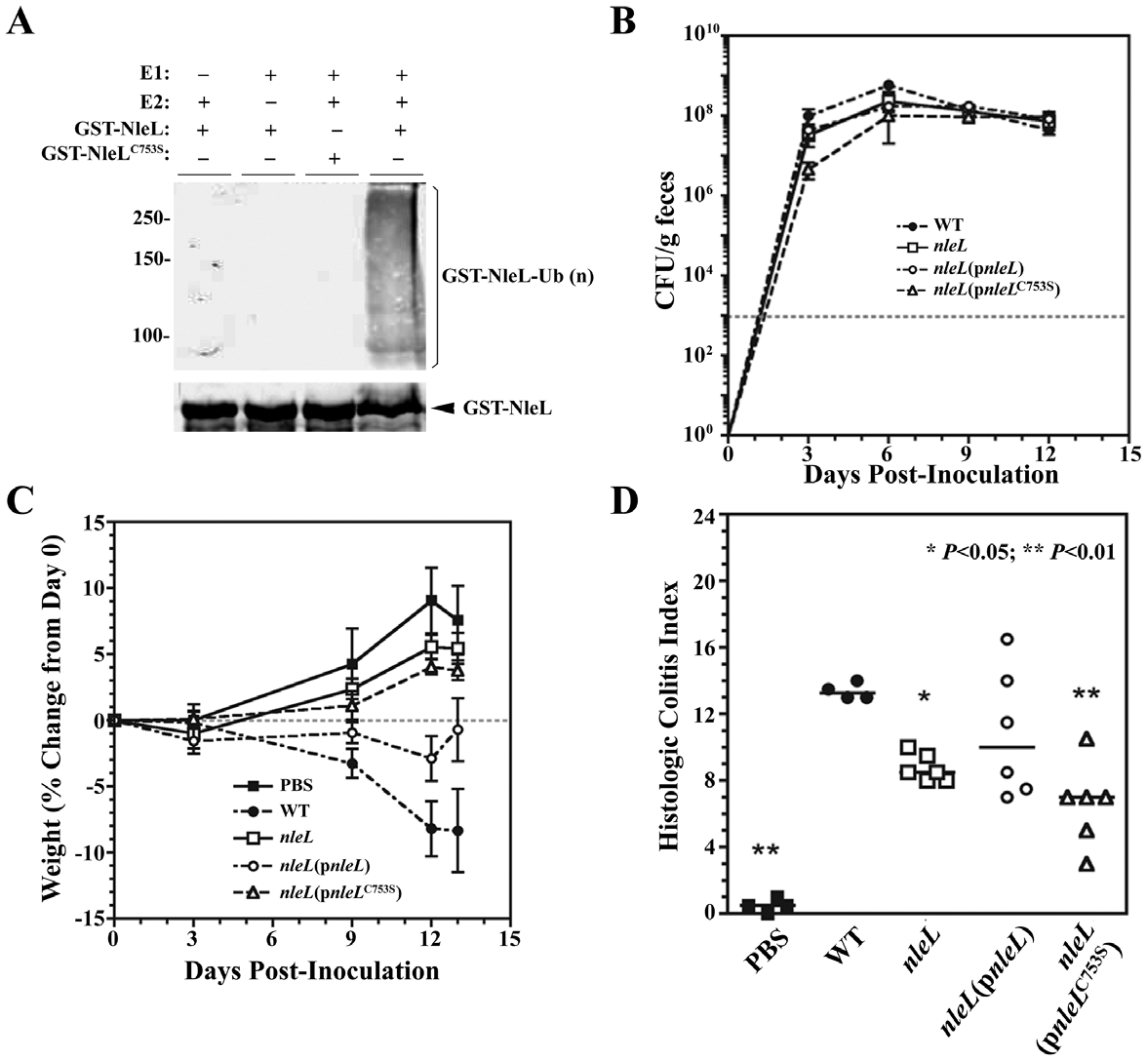
Contribution of *nleL* to *C. rodentium* virulence. (**A**) *C. rodentium* NleL is an E3 ubiquitin ligase. Combinations of ATP, ubiquitin, E1, UbcH5a and GST-NleL or GST-NleL^C753S^ were incubated at 35°C for 90 min, and the Western blot was performed using polyclonal anti-ubiquitin antibodies (top) or anti-GST antibodies (bottom). (**B)** Fecal bacteria shedding of the wild-type strain (DBS130), the *nleL* null mutant strain, the *nleL* mutant strain expressing the wild-type NleL, or the *nleL* mutant strain complemented with the catalytically-dead NleL^C753S^ in *C. rodentium*. (**C**) Virulence of *C. rodentium* strains as indicated by weight of the mice post-inoculation. The percent weight change of the mice over the 13 days post-inoculation was measured. The error bars indicate standard errors. Mice infected with *nleL* deletion strain (DBS792) had significantly reduced weight loss when compared to the wild type (DBS130). This could be partially rescued by plasmid expressing the full length NleL (DBS793) but not by plasmid expressing the catalytically inactive NleL^C753S^ mutant (DBS794). (**D**) Contribution of *nleL* to mouse colitis caused by *C. rodentium*. Pathology of mouse colon by different *C. rodentium* strains, as indicated by histologic activity index (sum of lesion scores). At day 13 post-inoculation colon tissue was scored for lesions: inflammation, edema, epithelial defects, crypt atrophy, hyperplasia, and dysplasia. Mice infected with *nleL* deletion strain showed significantly reduced lesions when compared to the wild type (P<0.05). This could be rescued by plasmid expressing the full length NleL but not by plasmid expressing the catalytically inactive NleL^C753S^ mutant (P<0.01).

The efficiency of pedestal formation may influence the bacterial colonization and/or degree of inflammation during natural infection. *C. rodentium* has been used as a model microorganism to assess the virulence of the A/E pathogens in the mouse infection model. To evaluate whether NleL plays a role in an animal model, we first determined if *C. rodentium* NleL has E3 ligase activity in an *in vitro* ubiquitination assay using the *in vitro* auto-ubiquitination assay. GST-NleL^59–782^ was added as the potential E3 ligase. Western blotting with either GST or ubiquitin antiserum shows the presence of mono and poly-ubiquitinated *C. rodentium* NleL (**Fig. 5A**). No ubiquitination of *C. rodentium* NleL was observed when GST-NleL^C753S^ was used or in the absence of E1 or UbcH5a indicating that each of these components of the ubiquitination reaction is required for the ubiquitination of NleL. This indicates that *C. rodentium* NleL is an E3 ubiquitin ligase and C753 is essential for the ligase activity. We next determined if *C. rodentium* NleL plays a role in *C. rodentium* virulence in a murine infection model.

*C. rodentium* infection in C57BL/6 mice is characterized by loose stool progressing to diarrhea in severe cases, poor overall body condition, and weight loss, which can be fatal particularly in young animals [50], [51]. Colonic lesions consist of epithelial hyperplasia, submucosal edema, mucosal erosion and ulceration, and inflammatory infiltration varying from submucosal to transmural [50], [52]. As shown in **Fig. 5B**, fecal bacteria shedding of the wild-type strain (DBS130) of *C. rodentium* reached a maximum at 6 days post-inoculation (DPI) of 6×10^8^ CFU/g of feces in agreement with previous reports of *C. rodentium* infection of C57BL/6J mice [52], [53], [54]. Fecal shedding of the *C. rodentium nleL* null mutant strain (DBS792), the *nleL* mutant strain expressing the wild-type *C. rodentium* NleL (DBS793), or the *nleL* mutant strain complemented with the catalytically-dead NleL^C753S^ (DBS794) were comparable to the wild-type *C. rodentium* by 6 DPI and remained comparable until sacrifice at 13 DPI (**Fig. 5B** **)**.

In spite of the observation that NleL had no significant effect on colonization, this effector promoted disease. By 12 DPI uninoculated mice had gained 8% of their initial body weight, whereas mice inoculated with either the wild-type or *nleL* complemented strain had significant weight loss (8% and 3% of initial weight by 12 DPI, *P*<0.001, **Fig. 5C**). In contrast, mice inoculated with the *nleL* mutant or the non-complementing point mutant NleL^C753S^ gained weight (5% and 4%, respectively) and were not significantly different from uninoculated mice throughout the study. In addition, infection with wild-type *C. rodentium* resulted in robust colitis with multifocal erosions, infiltration of neutrophils, lymphocytes and macrophages, hyperplasia, and herniation of glands into local lymphoid tissue. At 13 DPI the histologic colitis index was comparable between mice inoculated with either wild-type strain (median index of 13.25 [13.0 to 14.0]) or *nleL-*complemented strain (10.0 [7.0 to 16.5]) of *C. rodentium* (**Fig. 5D**). Deletion of *nleL* from *C. rodentium* resulted in reduced lesion severity and colitis in mice (8.5 [8.0–10.0]) compared to wild-type inoculated mice (P<0.05). Similarly, inoculation with the non-complementing NleL^C753S^ point mutant resulted in less severe colitis than wild-type *C. rodentium* (P<0.01) with a histologic colitis index of 7.0 (5.0–10.5) that was statistically indistinguishable from the *nleL* mutant.

## Discussion

EHEC and EPEC are members of the Attaching and Effacing pathogen group of organisms that cause serious food- and water-borne illnesses in humans. A pathogenic infection by EHEC or EPEC involves attachment of the pathogen to the surface of the host cell followed by translocation of key effector proteins into the cytoplasm via the TTSS. Although the molecular events that follow differ somewhat between EHEC and EPEC, they result in the rearrangements of host cytoskeletal machinery, which in turn leads to the formation of morphologically similar pedestals, a hallmark of an A/E pathogenic infection. Although a considerable amount of research on the mechanism of pedestal formation has been carried out, little is known about how this process is regulated. We demonstrated here that EHEC utilize the host ubiquitination pathway to down-regulate the pedestal formation, perhaps to maintain a balance to co-exist with the host cells. Thus, the bacteria is involved actively not only in the formation, but also the modulation of pedestal formation. Consistent with the hypothesis that unregulated pedestal formation may alter disease, we demonstrate the importance of NleL and its E3 ubiquitin ligase activity in development of clinical and pathological disease caused by *C. rodentium*.

EHEC NleL shares sequence similarity to *Salmonella* SopA mainly toward the C-terminus where the E3 ligase activity lies [28], [30]. A recent study found that both NleL and SopA form the bilobed catalytic domain reminiscent of the N- and C-lobe architecture of HECT E3 ligases [45]. Although both NleL and SopA possess poly-ubiquitination activities *in vitro*, NleL form free (unanchored) poly-ubiquitin chains when the GST-free NleL is used in the ubiquitination assay [45]. *Salmonella* is able to invade the host cells and survive inside the host cells. In contrary, EHEC exerts its virulence by adhering to the surface of the host cells via actin-rich pedestals. It is not clear whether the different biochemical E3 ligase activities contribute to the different life-style of the two intestinal pathogens. We speculate that NleL may exploit the free ubiquitin pools in the host cell to exert its function without the canonical substrates. Further studies are needed to examine this possibility.

Our data demonstrate that loss of the NleL E3 ligase activity leads to increased pedestal formation. Preliminary work showed that the loss of the NleL E3 ligase activity did not alter the expression of Tir or EspFu (**Fig. 2A** and data not shown), suggesting that NleL may exert its function after their translocation. EPEC infection has been shown to lead to pedestal formation much more efficiently than that of EHEC *in vitro* [55]. A significant difference between the two pathogenic types lies in the nature of the involvement of Tir during pedestal formation. While Tir_EPEC_ is tyrosine phosphorylated upon entry into the host cell and interacts with host adaptor protein Nck, which stimulates Arp2/3-mediated actin polymerization [49], [56], Tir_EHEC_ lacks the homologous tyr residue, Y474, and depends on another effector EspFu/TccP to induce actin polymerization [9], [10], [23]. Such studies highlight the differences in the mechanism of pedestal formation between the two organisms. Our data indicate that NleL probably functions at steps common to EPEC and EHEC-mediated pedestal formation. It is reasonable to speculate that NleL ubiquitinates an unknown factor of bacterial or host origin involved in pedestal formation. One such scenario would be that host cell proteins involved in pedestal formation are ubiquitinated by NleL which, in turn, corresponds to a decrease in the level of host proteins or alters their localization, resulting in a decrease in pedestal formation. Alternatively, NleL may ubiquitinate Tir and promote its endocytosis, thus decreasing the surface availability of Tir. Our preliminary studies have shown that Tir is not a substrate for the E3 ligase activity of NleL in an *in vitro* ubiquitination assay (data not shown). However, we cannot rule out that a third factor is required, but absent, in our *in vitro* reaction. A recent structural study showed that another non-LEE-encoded effector, NleG, is a RING finger ubiquitin E3 ligase [57]. It is tempting to speculate that NleL, NleG and Tir may work together to modulate the actin pedestal formation. In addition, Tir is inserted into the plasma membrane of the host cell in a hairpin loop structure. It is also possible that NleL is affecting Tir localization. Further studies are required to dissect the exact molecular mechanism on how the E3 ligase activity of NleL modulates the pedestal formation.

## Materials and Methods

### Bacterial strains, plasmids and mammalian cell lines

The Enterohemorrhagic strain designated *E. coli* O157:H7 (ZP250), a spontaneous nalidixic acid mutant of an outbreak strain isolated from apple juice (RM1484) [58], [59]. Using ZP250 as a parent, strains harboring in-frame chromosomal deletions of genes *escF* (ZP251) were generated with the help of an allelic-exchange suicide vector as described previously [60]. The EHEC strain expressing mutant NleL^C753A^ (ZP254) was generated similarly using a plasmid encoding the NleL^C753A^ (pZP2111). *C. rodentium nleL* null deletion strain was similarly generated. Complementing strains were created by introducing the plasmid expressing the wild-type *C. rodentium* NleL (pZP1661) or the catalytically-dead NleL^C753S^ (pZP1664) into DBS792 generating DBS793 and DBS794 respectively. The EHEC *nleL*-M45 complementation plasmid (pZP1666) was constructed by cloning DNA fragments encoding full length NleL and its promoter sequence (250 bp upstream of the translation start site) into pSB1136, a pBAD derivative [61]. The *nleL* translocation plasmid (pZP1671) was subcloned by ligating *nleL* DNA sequences from the complementation plasmid with appropriately digested *cyaA* reporter gene plasmid (pZP599) [62]. The HA-tagged EHEC Tir complementation was previously described [9].

The plasmid expressing amino acid residues 59 through 782 of NleL (NleL^59–782^) as a fusion protein with glutathione S-transferase (GST) was constructed by subcloning the relevant *nleL* coding sequence into pGEX-KG (pZP1173) [63]. Single base substitution mutations changing residues either C688 (pZP1667) or C753 (pZP1668) to Serine or Alanine were introduced using the Quick-Change Site-Directed Mutagenesis Kit (Stratagene, La Jolla, CA) according to the manufacturer's protocol. The plasmid expressing C-terminal 6xHis-epitope tagged Tir (pZP1517) was constructed by cloning Tir coding sequence into pET28a (EMD Biosciences, Madison, WI).

HeLa cells from ATCC Cell Biology Stock Center (Manassas, VA), were grown in Dulbecco's modified Eagle's medium supplemented with 10% fetal calf serum (Gibco, Carlsbad, CA, USA).

### Purification of recombinant proteins from *E. coli*

Recombinant fusion proteins, GST-NleL and Tir-6XHis were purified from *E. coli* BL21(DE3) harboring respective plasmids using glutathione- Sepharose 4B (Amersham Biosciences, Piscataway, NJ) and Ni-NTA resin (Qiagen, Valencia, CA), respectively. All purified proteins were dialyzed and resuspended in phosphate-buffered saline (PBS) containing 2 mM dithiothreitol.

### Bacterial infection

EHEC strains were grown overnight standing at 37°C in Luria-Bertani (LB) broth supplemented with nalidixic acid (50 µg ml^−1^) and used directly for infection [20]. EPEC strains were grown overnight standing at 37°C in LB supplemented with followed by subculture (1∶100) in Hepes-buffered DMEM until optical density of the cultures measured at 600 nm reached 0.7 [9]. Cultures were then used to infect HeLa cells grown in 24-well tissue culture plates at 37°C in 5% CO_2_ with 10% fetal calf serum (Gibco, Carlsbad, CA, USA), at a multiplicity of infection (moi) of 100, unless specified otherwise.

### Protein secretion assay

Bacterial strains were grown overnight in LB at 37°C. The cultures were then diluted 1∶20 in M-9 minimal media supplemented with 0.4% glucose, 8 mM MgSO_4_, 44 mM NaHCO_2_, and 0.1% Casamino Acids and grown standing at 37°C in 5% CO_2_ until the OD_600_ reached approximately 0.8. The bacterial cultures were centrifuged and the supernatants were filtered through a 0.22 um filter to remove any residual bacteria. Proteins in the supernatants were precipitated with 10% trichloroacetic acid on ice for at least 1 hour. Precipitated proteins were collected by centrifugation and washed twice with ice-cold acetone before being resuspended in SDS-PAGE loading buffer.

### Adenylate cyclase translocation assay

HeLa cells were infected as described. Four hours post-infection, cells were washed with ice-cold PBS and lysed using 0.1 M HCl with gentle agitation for 20 min. Protein concentration in cell lysates was determined using the Bio-Rad Protein Assay Kit (Hercules, CA) according to the manufacturer's instructions. Equal amount of protein was used to determine levels of Adenosine 3′, 5′-cyclic monophosphate (cAMP) using the Direct Immunoassay Kit (Assay Designs, MI) according to the manufacturer's instructions. Adenylate cyclase activity is expressed as pmol per milligram of total protein.

### Immunofluorescence microscopy

HeLa cells infected for 3 hours were maintained in fresh media for an additional 3 hours. Cells were washed with cold PBS, fixed in 3% formaldehyde and permeabilized with 0.1% Triton X-100 [4]. Cells were incubated with either rabbit anti-O antibody (EHEC O157, Difco Laboratories, Detroit, MI; EPEC O111, Denka Seiken Co., Tokyo, Japan) as primary and Alexa Flour 488 conjugated anti-rabbit (1∶300 dilution) secondary antibody or Texas-red Phalloidin (1∶300; Molecular Probes, Carlsbad, CA). All images represent black and white projections of *z*-section slices obtained on a Zeiss LSM 700 confocal microscope. Approximately 300 cells were counted from each infection. Based on the number of pedestals per cluster, the clusters were divided into groups; extra large cluster (>20 pedestals/cluster for EPEC), large clusters (>10 pedestals/cluster), medium clusters (6–10 pedestals/cluster) and small clusters (1–5 pedestals/cluster).

### *In vitro* ubiquitination assay

*In vitro* ubiquitination experiments were carried out as described [64]. Briefly, a reaction mixture containing 40 mM Tris-HCl, pH 7.5, 5 mM MgCl_2_, 2 mM ATP, 2 mM dithiothreitol, 300 ng/µl ubiquitin [65], 0.1 µM E1 activating enzyme, 0.5 µM UbcH5a, E2 ubiquitin-conjugating enzyme (Boston Biochem, Boston, MA), 2 µg GST-NleL, GST-NleL^C688S^, GST-NleL^C753S^, or GST-NleL^C753A^ were incubated at 35°C for 90 min and subjected to SDS-PAGE (8%) and Western blot analysis using anti-GST or anti-MBP antibodies (New England Biolabs, Ipswich, MA). A similar *in vitro* ubiquitination assay was performed for each of the E2 ubiquitin-conjugating enzymes of interest: UbcH2, UbcH3, UbcH5a, UbcH5b, UbcH5c, UbcH6, UbcH7 and UbcH10 (Boston Biochem, Boston, MA).

### Mice Infection

Female 5-week old C57BL/6J (The Jackson Laboratory, Bar Harbor, Maine) mice were fed a rodent diet and water *ad libitum* and housed in micro-isolator cages that were maintained specific-pathogen-free of known murine bacterial, viral and parasitic infections including all known *Helicobacter* spp. in facilities at MIT approved by the Association for Assessment and Accreditation of Laboratory Animal Care, International. Mice were divided into five groups and gavaged with ∼2×10^9^ of an overnight culture of the wild-type *C. rodentium* DBS130 (n = 4), the *nleL* null mutant strain DBS792 (n = 6), the *nleL* mutant strain expressing the wild-type NleL DBS793 (n = 6), or the *nleL* mutant strain complemented with the catalytically-dead NleL^C753S^ DBS794 (n = 6) in 100 µl PBS. Uninoculated mice were gavaged with 100 µl sterile PBS (n = 4). *C. rodentium* fecal shedding was monitored by serial dilution plating of fecal slurries on MacConkey agar with selection for nalidixic acid or chloramphenicol. Body weights of individual mice were monitored and mice sacrificed at 2 WPI. At necropsy, colon was collected, fixed in 10% formalin, paraffin embedded, sectioned at 5 µm, and stained with hemoxylin and eosin for histologic evaluation. Colonic tissue sections were scored on a scale of 0–4 (where 0  =  no lesion, 1  =  minimal, 2  =  mild, 3  =  moderate, and 4  =  severe) for inflammation, edema, hyperplasia, dysplasia, epithelial defects, and crypt atrophy by a board-certified blinded pathologist. Lesion scores are presented as histologic activity indices that are a sum of all six categorical scores (maximum of 24). Bacterial shedding and weight change were analyzed by two-way ANOVA with Bonferroni post-test. Histologic colitis indices were evaluated by one-way ANOVA with Dunnett's post-test compared to DBS130.

### Ethics Statement

This study was carried out in strict accordance with the recommendations in the Guide for the Care and Use of Laboratory Animals of the National Institutes of Health. The protocol was approved by the Animal Care and Use Committee at the Massachusetts Institute of Technology (Protocol Number: 0207-020-10). All efforts were made to minimize suffering.
